# Excitatory and inhibitory lateral interactions effects on contrast detection are modulated by tRNS

**DOI:** 10.1038/s41598-019-55602-z

**Published:** 2019-12-17

**Authors:** L. Battaglini, G. Contemori, A. Fertonani, C. Miniussi, A. Coccaro, C. Casco

**Affiliations:** 1 Department of General Psychology, University of Padova, Padova, Italy; 2 Neuro.Vis.U.S. Laboratory, University of Padova, Padova, Italy; 3 Université de Toulouse-UPS, Centre de Recherche Cerveau et Cognition, Toulouse, France; 4 Cognitive Neuroscience Section, IRCCS Istituto Centro San Giovanni di Dio Fatebenefratelli, Brescia, Italy; 5 Center for Mind/Brain Sciences - CIMeC, University of Trento, Rovereto, Italy

**Keywords:** Perception, Pattern vision

## Abstract

Contrast sensitivity for a Gabor signal is affected by collinear high-contrast Gabor flankers. The flankers reduce (inhibitory effect) or increase (facilitatory effect) sensitivity, at short (2λ) and intermediate (6λ) target-to-flanker separation respectively. We investigated whether these inhibitory/facilitatory sensitivity effects are modulated by transcranial random noise stimulation (tRNS) applied to the occipital and frontal cortex of human observers during task performance. Signal detection theory was used to measure sensitivity (*d*’) and the Criterion (C) in a contrast detection task, performed with sham or tRNS applied over the occipital or the frontal cortex. After occipital stimulation results show a tRNS-dependent increased sensitivity for the single Gabor signal of low but not high contrast. Moreover, results suggest a dissociation of the tRNS effect when the Gabor signal is presented with the flankers, consisting in a general increased sensitivity at 2λ where the flankers had an inhibitory effect (reduction of inhibition) and a decreased sensitivity at 6λ where the flankers had a facilitatory effect on the Gabor signal (reduction of facilitation). After a frontal stimulation, no specific effect of the tRNS was found. We account for these complex interactions between tRNS and flankers by assuming that tRNS not only enhances feedforward input from the Gabor signal to the cortex, but also enhances the excitatory or inhibitory lateral intracortical input from the flankers. The boosted lateral input depends on the excitation-inhibition (E/I) ratio, namely when the lateral input is weak, it is boosted by tRNS with consequent modification of the contrast-dependent E/I ratio.

## Introduction

Visual performance for a stimulus presented in a given retinal location can be modulated by the simultaneous presence of other stimuli having a different retinal position. This technique, known as lateral masking, consists in measuring contrast sensitivity for a periodic Gabor pattern (target) flanked by high-contrast Gabors collinear and iso-oriented to the target. Psychophysical studies on lateral masking showed that in central vision sensitivity reduces (threshold increases) when the distance from the flankers is ≤2 target wavelengths, a result suggesting lateral inhibition by the flankers. For larger target-to-flankers distances, ranging from 3 to 9 target wavelengths, the flankers facilitate target detection, as demonstrated by a threshold decrease from absolute threshold^1–8^. Indeed, Mizobe and colleagues^9^ not only showed that the neurometric function to target contrast was modulated by the flankers presented outside the classical receptive field, but also that the modulation was dependent on the relative distance between target and flankers. Moreover, the separations at which facilitation occurs is larger in the periphery than in the fovea^7^. Furthermore, psychophysical^4,5,8^, electrophysiological^10^, and brain imaging studies^11^ showed that the polarity of contextual modulation is also contrast dependent: inhibitory effects occur within a contrast range larger than that at which facilitation occurs.

One major question regards the neurophysiological bases of the facilitatory and inhibitory lateral interactions. Psychophysical evidence suggests that detection thresholds depend on the activation of interconnected local neural network with both excitatory (E) and inhibitory (I) neurons whose synaptic connections are activity dependent. E/I ratio depends on the contrast of the target and on the E and I lateral input that may favor either facilitation or inhibition by the flankers depending on target-flanker separation^3^.

Contextual influences on contrast detection have been investigated in an accumulating mass of studies for two crucial reasons. First, they are considered to contribute to the perception of contours in natural scenes. Facilitation of detection occurs when the target-flanker configuration is collinear rather than orthogonal, that is consistent with a contour structure in which local global orientation cohere^12–14^. Many authors were refrained from drawing a too close parallel between lateral masking and supra-threshold perceptual phenomena^15^. Mechanisms involved in suprathreshold perceptual tasks, such as contour integration and crowding, do not use any simple form of local contrast enhancement to perform grouping and segmentation of local elements, respectively. However, even assuming that contrast enhancement may not be the mechanism involved in either perceptual grouping or segmentation, it is quite likely that these high-level tasks and the low-level effects of contrast enhancement could be explained by a common cortical circuit^7,13,16^. The second reason for which contextual influences in contrast detection have caught the attention of several investigations in the last decades is that, when they are made inefficient by a visual disorder they can be partially restored by promoting, through training, neural plasticity at the level of lateral intracortical connections in V1. Thus, modulation of intracortical connections may result in a powerful rehabilitation tool for low vision patients. Most studies have used perceptual learning to induce neural plasticity in normal and in low vision population^17–24^.

Neural plasticity induction can be also achieved through weak currents applied transcranially. The most used of these protocols are: transcranial direct current (tDCS), alternating (tACS) and random noise (tRNS) stimulation. Whereas tACS has been suggested to be suitable for interacting with endogenous brain oscillations^25–29^, tDCS, and tRNS have become increasingly popular as tools to induce neural modulation in the visual system^30–33^. With tDCS that is a constant current, in general, the anodal electrode is associated with an increase in excitability, while an inhibitory effect is observed with the cathodal electrode^34^. Instead, with tRNS the direction of the current is not relevant to obtain effects^35^. In this framework tRNS similarly to anodal tDCS, has been shown to induce an increase of cortical excitability^36^ but likely with a different dynamic^32^ avoiding inactivation due to adaptation of ion channels when using a constant current. In contrast to anodal tDCS, it has been hypothesized that tRNS prevents homeostasis of the system. Such stimulation consists in the application of a random electrical oscillation spectrum over the cortex; this fast oscillating field modifies the neurons’ synaptic efficiency regardless of the current flow orientation^35,36^. Mechanistically, tRNS-induced neurophysiological effect has been suggested to originate from modulation of voltage-gated sodium channels^32,36^ specifically acting on the dynamics of in/activation of the sodium channels^37^. Moreover, the behavioral improvement following tRNS has been interpreted by suggesting that the random frequency stimulation produced by tRNS sustains random neural activity in the system, i.e., noise, which serve as a pedestal to expand the sensitivity of the neurons to weak stimuli, providing in same cases inputs similar to those of the target, thereby increasing the signal-to-noise ratio^38^. When applied over visual areas, tRNS increases perceived contrast of targets having low contrast^39^. In line with this approach, an intriguing possibility that better defines neurophysiological mechanisms is that, with lateral masking configurations, tRNS might induce a synaptic enhancement at the level of the lateral connections between target and flanker neurons, by inducing a temporal summation of weak depolarizing currents. Hence, specific changes in performance are related to a network-dependent stochastic resonance phenomenon^40^ i.e., the balance between excitation and inhibition is strictly related to the specific neuronal population state (E/I) and not just to generalized changes in cortical excitability. In this study, because these evidences that tRNS exceeds the beneficial advantages of tDCS, we aimed to explore if the interaction between tRNS and visual system task dependent activity can modulate cortex excitability and therefore behaviour in a specific way. Depending on which of the two circuitries is involved, either the one accounting for increase in perceived contrast for the target or the one responsible for the modulation of target contrast by lateral interactions, a different perceptual outcome is expected. A simple contrast gain effect would be reflected in an increase in sensitivity (*d*’) for a single target of low contrast and modulate the lateral interactions effect consequently. Based on the evidence that tRNS depolarize neurons, we expected tRNS to increase the E thalamic input only when this is weak, that is when the target contrast is low. In this case, given the evidence that E and I lateral modulation occur when the target contrast is low and high respectively^4,6,8,10^ we expected the E lateral input to be weakened with tRNS. That is, the reduced strength of lateral input, as reflected into a reduced facilitation by the flankers, would be an epiphenomenon of the change of contrast gain^41^. Alternatively, tRNS effect might be dependent on target-to-flankers distance and reflect a direct modulation of the relative strength of E or I lateral input from the flankers to a target, depending on which is weaker. In this case, we might expect an effect of tRNS even if tRNS has no effect at all on target perceived contrast.

## Method

### Observers

In total 68 young subjects participated in this study (46 females; mean age 24 ± 3 years). All participants had normal or corrected-to-normal vision and were naïve as to the purpose of the experiments. Thirty-eight participants were involved in the main Experiments 1 (N = 19; 15 females; mean age 24 ± 4) and 2 (N = 19; 12 females; mean age 24 ± 4) and 30 participants were involved in the Control Experiments 3 (N = 15; 10 females; mean age 24 ± 2) and 4 (N = 15; 9 females; mean age 24 ± 3). Participants in Experiment 1 and 3 were tested with flankers distant from the target, whereas participants in Experiment 2 and 4, were tested with the flankers close to the target. All participants took part voluntarily and informed consent was obtained from all participants before the study initiated. The study conformed to the tenets of the Declaration of Helsinki and the experimental methods have approval from Ethical Committee of the University of Padova (protocol 1719).

### Apparatus

Stimuli were displayed on a 22-in. Philips 202P4 CRT monitor with a refresh rate of 85 Hz. The minimum and maximum luminance of the screen were 0.6 and 112.1 cd/m^2^, respectively, and the mean luminance was 56.8 cd/m^2^. Luminance was measured with a CRS Optical photometer (OP200-E; Cambridge Research System Ltd., Rochester, Kent, UK). A digital-to-analog converter (Bits#, Cambridge Research Systems, Cambridge, UK) was used to increase the dynamic contrast range (12-bit luminance resolution). A 12-bit gamma-corrected lookup table (LUT) was applied so that luminance was a linear function of the digital representation of the image. The screen resolution was 1600 × 1200 pixels.

### Stimuli

**Figure 1:**
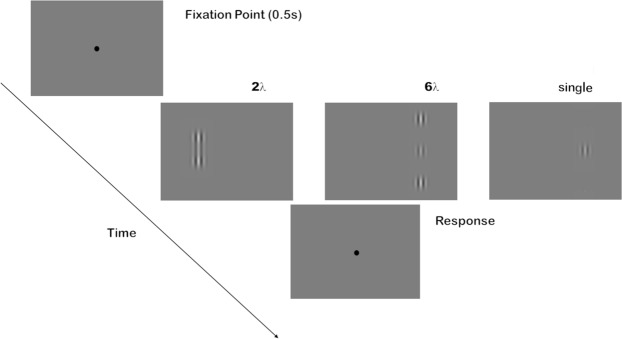
The stimulus configuration used in the experiments. Left to right: target-flanker configuration at 2λ, 6λ and single Gabor target.

Stimuli were generated using Matlab Psychtoolbox^42,43^. They were formed by a vertical Gabor target patch and, when present, by two collinear Gabor flankers (Fig. 1). Each Gabor patch consisted of a cosinusoidal carrier enveloped by a stationary Gaussian (Eq. 1) and was characterized by its sinusoidal wavelength (λ), phase (*φ*), and standard deviation of the luminance Gaussian envelope (σ) in the (x, y) space of the image:

1 $$G(x,y)=\,\cos (\frac{2\pi }{\lambda }x+\varphi ){e}^{(-\frac{{x}^{2}+{y}^{2}}{{\sigma }^{2}})}$$

with σ = λ and *φ* = 0 (even symmetric). Gabor patches had a spatial frequency of 1 cycle per degree (c/deg). Target-to-flankers distance was 6λ (wavelengths distance) in Experiment 1 and 3 and 2λ in Experiment 2 and 4. Since the thresholds for contrast differ between 6λ and 2λ, in order to sample from the floor to the ceiling we had to adopt two different ranges of contrasts in the two experiments. Each range has been derived from the literature^2,4^ and then adjusted with a pilot experiment. They varied according to eight levels: 0, 0.0020, 0.0028, 0.0039, 0.0055, 0.0077, 0.0105, 0.0150 in Experiment 1 and 3 and 0, 0.0030, 0.0058, 0.0110, 0.0250, 0.0410, 0.0800, 0.3000 in Experiment 2 and 4 (see Table 1). The contrast of the flankers was fixed at 0.6.

**Table 1:** To promote a suppressive effect of the flankers placed at short distances from the target (2λ), we used a relatively higher range of contrasts with respect to the 6λ distance.

Contrast											
	Very low	low							medium		
6λ	0.002	0.0028	0.0039	0.0055	0.0077	0.01005	0.015				
2λ		0.003		0.0058		0.011		0.025	0.041	0.08	0.3

Note however, that certain contrast levels in the range .003 to .011 were the same in the two λ distances. This allowed us to isolate a specific effect of the flankers, independently on target contrast.

### Procedure

Observers sat in a dark room at a distance of 57 cm from the screen. Viewing was binocular. Stimuli appeared randomly for 100 ms to the left or to the right of fixation. The distance from the center of the screen to the center of the Gabor configuration was 4 deg. Observers were required to maintain fixation on the central fixation mark, which was always present except during stimulus presentation, to provide a transient cue for advising observers that the stimulus was present even if, at low contrast, they could not detect it. Observers performed a yes-no task in which they were asked to report whether they could perceive the central target by pressing the response key. The next trial started after 0.5 s from the response keypress. Each experiment was devoted to one target-to-flankers distance (either 2λ or 6λ) and comprised a repetition of two sessions, each one consisting of 112 trials: 8 target contrast levels × 2 stimulus positions × 7 repetitions. In the first session, the target was flanked by collinear Gabor patches, in the second one, the target was presented alone. Participants performed the two sessions twice, once while they received Sham stimulation and once while they received tRNS. The order of the two configurations sessions was the same in the Sham and tRNS session, but it was counterbalanced across participants. The order of stimulation (Sham vs. tRNS) was also counterbalanced across participants in order to avoid a possible tRNS dependent after-effects.

### tRNS

A battery-driven current stimulator (BrainStim, EMS, Bologna, Italy) delivered high-frequency tRNS through a pair of conductive rubber electrodes inserted in a 5 by 7-cm physiological solution-soaked synthetic sponge. The tRNS consisted of a randomly alternating current of 1.5 mA with a 0 mA offset, whose frequency ranged from 100 to 600 Hz. In the main Experiments 1 and 2, the electrode of interest was placed over V1/V2 (Oz) and the other electrode over the vertex (Cz), as in previous studies^30,44,45^. The experiments 3 and 4 served as a control to test the spatial specificity of stimulation in producing its effect on the contrast gain for the target and/or on E and I lateral interactions. The only difference with the main experiments was the electrode montage: the electrode of interest was placed over the forehead (between Fpz and nasion) and the other electrode over the vertex (Cz). tRNS was applied for approximately 12 minutes. It was started at the onset of the first session, and it was stopped at the end of the second session, with no pause between the two experimental sessions. The Sham stimulation consisted of 30 seconds delivered only during the first session. The duration of the fade-in/fade-out period was 15 second for both tRNS and Sham stimulation. At the end of each experimental session we asked the participant to complete a sensation questionnaire^46^. Very few participants reported mild skin sensation at the onset of the stimulation, but it disappeared after few seconds. The guessing rate of real/placebo stimulation was at chance levels.

### Statistical analysis

To ascertain that the response to the single and flanked targets depended on contrast we pooled sensitivity (*d*’) of the person to the signal and on the Criterion, that is the cutoff value determined by the observer trying to detect the target, and regressed the pooled data against the contrast. *d*’ and Criterion were calculated according to Signal Detection Theory. Two-ways repeated measures ANOVAs were then used to analyze the changes in sensitivity (SC = *d*’_collinear_ − *d*’_single_) and the change in Criterion (CC = C_collinear_ − C_single_) due to the contextual modulation by the flankers on the target. The main factors were: Stimulation (Sham vs. tRNS) and Contrast levels (seven levels, see Stimuli section). Post-hoc pairwise comparisons were conducted using two-tailed t-tests with Bonferroni correction. Separated ANOVAs were conducted for the data of Experiment 1–3 (6λ) and Experiment 2–4 (2λ) because the contrast levels didn’t match for the two λs. One-tail t-tests based on the null hypothesis of 0 SC were also conducted to assess the polarity of contextual effects: SC > 0 indicated facilitation by the flankers were SC < 0 indicated inhibition.

## Results

### Main experiments results

**Figure 2:**
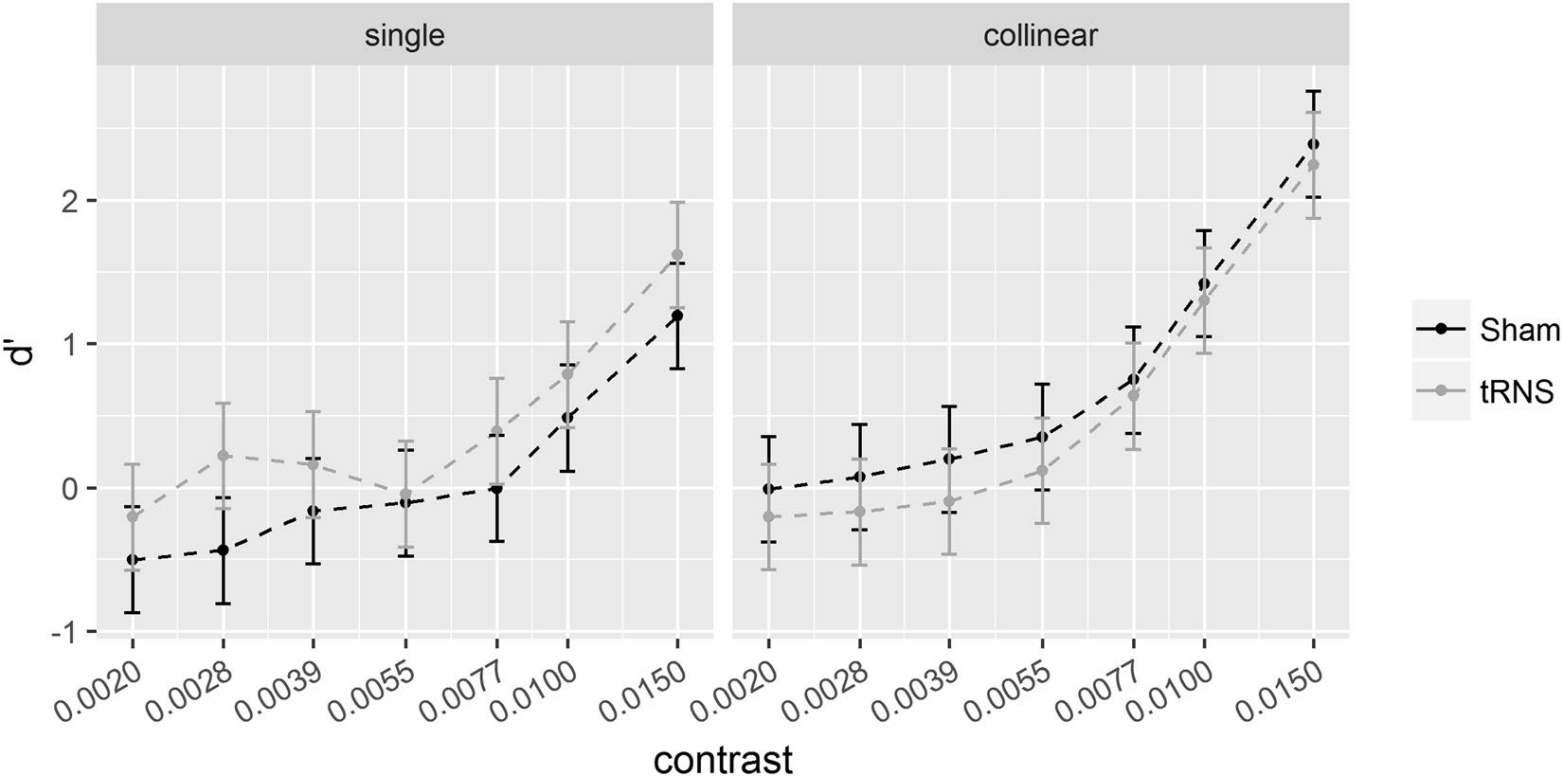
**6λ** configuration. Sensitivity (*d*’) for the single (left) and collinear target (right) is plotted as a function of target contrast separately for the Sham and tRNS sessions. Solid bars indicate Confidence Intervals (0.95%).

**Figure 3:**
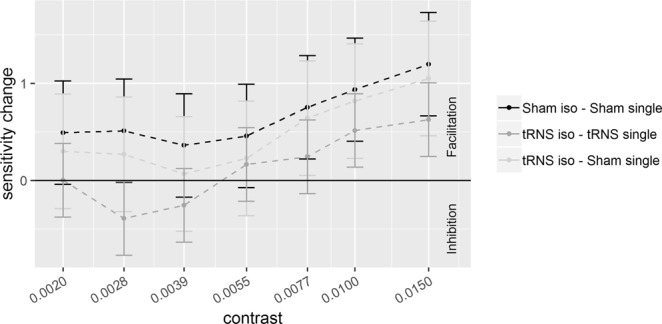
Sensitivity changes (SC), referring to the difference between *d*’ obtained in the collinear and single target (*d*’_collinear_ − *d*’_single_), are plotted as a function of target contrast with flankers at a distance of **6λ**. Positive values represent facilitation by collinear flankers whereas negative values represent inhibition. Solid bars indicate confidence interval (95%).

**Figure 4:**
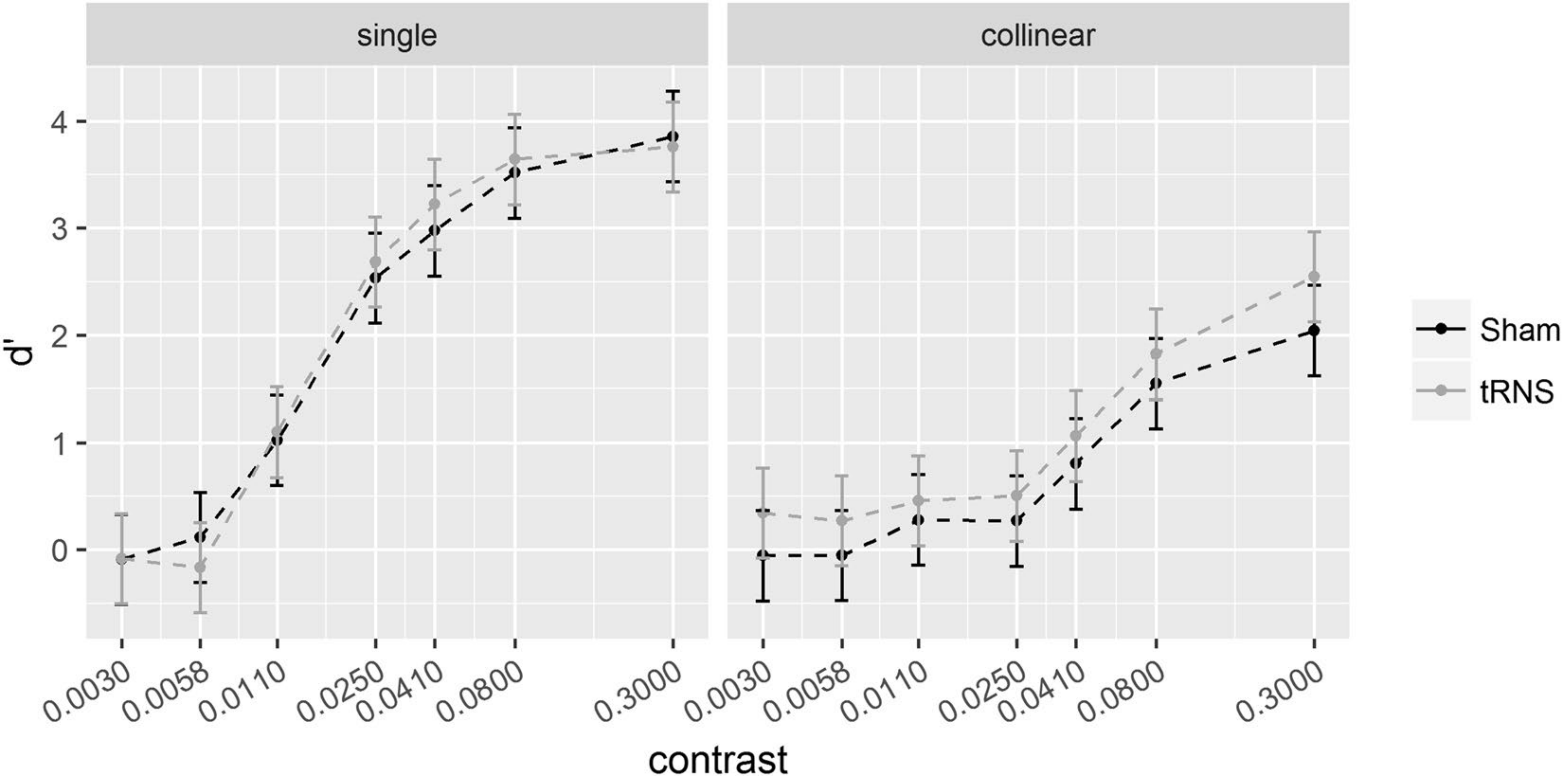
**2λ** configuration. Sensitivity (*d*’) for the single (left) and collinear target (right) is plotted as a function of target contrast, separately for the Sham and tRNS sessions. Solid bars indicate confidence interval (95%).

**Figure 5:**
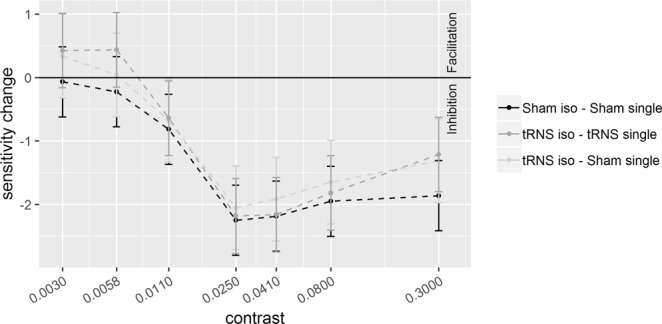
Sensitivity changes (SC), referring to the difference between *d*’ obtained in the collinear and single target (*d*’_collinear_ − *d*’_single_), are plotted as a function of target contrast with flankers at a distance of **2λ**. Positive values represent facilitation by collinear flankers whereas negative values represent inhibition. Solid bars indicate confidence interval (95%).

Results of Experiment 1 are illustrated in Fig. 2 (*d*’) and Fig. 3 (sensitivity change, SC = *d*’_collinear_ − *d*’_single_). Results of Experiment 2 are illustrated in Fig. 4 *(d*’) and Fig. 5 (SC).

**Figure 6:**
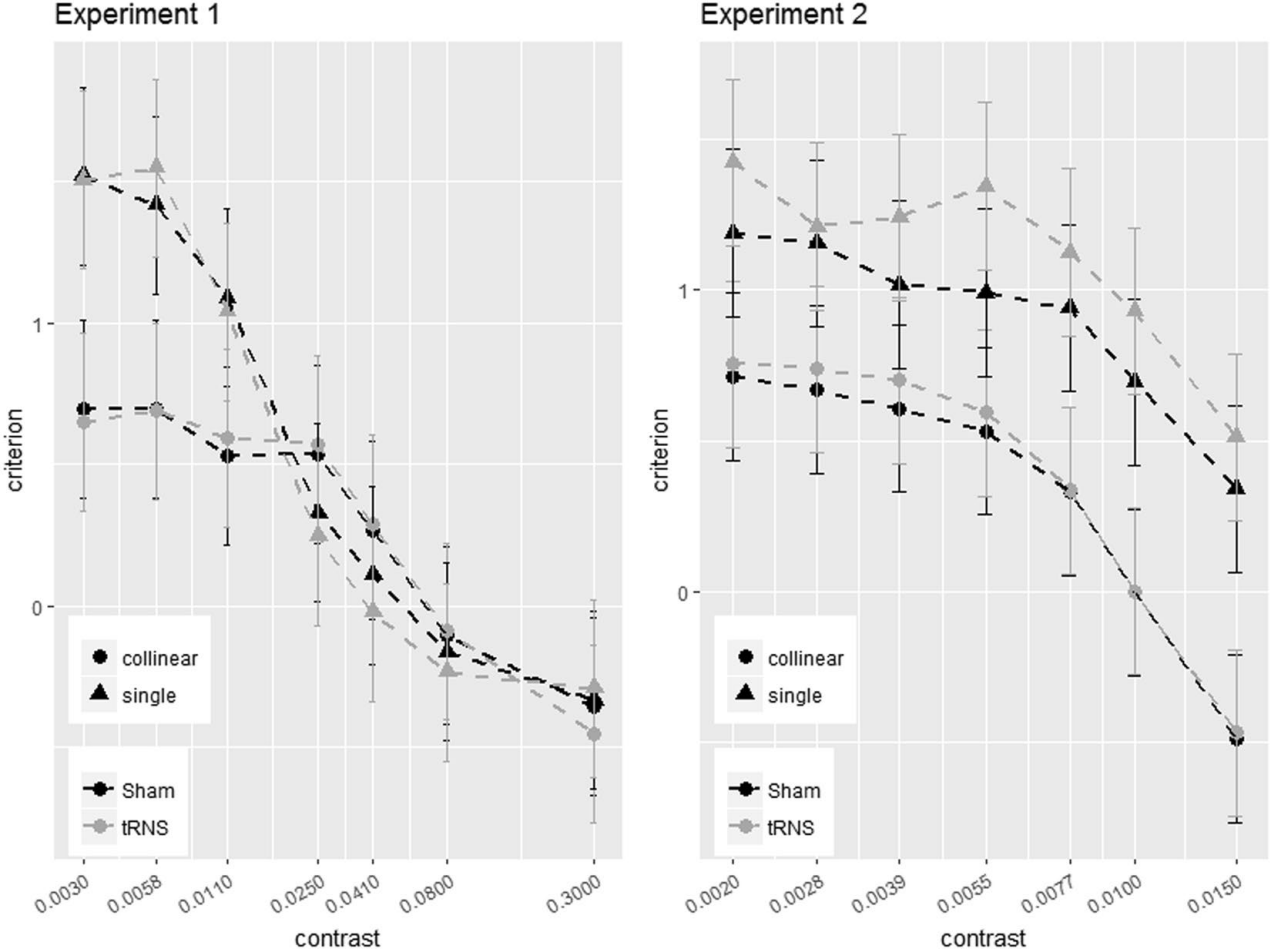
The figure shows the way the Criterion varies as a function of contrast in the two main experiments (Experiment 1, left panel; Experiment 2, right panel). In each panel, the Criterion C is shown for the single (triangle symbols) and collinear target (dot symbols) presented online with tRNS (grey broken lines) or with Sham (black broken lines). Solid bars indicate confidence interval (95%).

Figures 2 and 4 show the effects of tRNS on the sensitivity (*d*’) for the single target and collinear configuration. Figures 3 and 5 show SCs (*d*’_collinear_ − *d*’_single_). Figure 6 shows the Criterion results.

Pooled *d*’s correlated positively with contrast at 6λ (R^2^ = 0.64, *p* < 0.001) and 2λ (R^2^ = 0.46, *p* < 0.001), indicating higher sensitivity as contrast increases. The ANOVA on the SC revealed a significant effect of the Contrast Levels: SC became more positive (higher *d*’ in the collinear configuration) with increasing contrast at 6λ (F_(6,108)_ = 8.9, *p* < 0.001, η^2^_p_ = 0.33) and more negative (lower *d*’ in the collinear configuration) with increasing contrast at 2λ (F_(6,108)_ = 25.3, *p* < 0.001, η^2^_p_ = 0.58). Moreover, at 6λ (Experiment 1), the effect of the Stimulation is significant: tRNS reduced SC at 6λ (F_(1,18)_ = 5.9, *p* = 0.026, η^2^_p_ = 0.25) independently on the Contrast Levels (Stimulation × Contrast Levels: F_(6,108)_ = 0.74, *p* = 0.62, η^2^_p_ = 0.039). At 2λ on the other hand, an overall effect of tRNS on SC (reduction of inhibition) was not found (Stimulation: F_(1,18)_ = 2.48, *p* = 0.13, η^2^_p_ = 0.12) whereas the Stimulation × Contrast Levels interaction was significant (F_(6,108)_ = 2.21, *p* = 0.047, η^2^_p_ = 0.11); this indicates that tRNS reduced inhibition, non-significantly in the first (from -0.07 to 0.42, *p* = 0.085), and significantly in the second (from -0.23 to 0.44, *p* = 0.015) and in the highest level of contrast (from −1.86 to −1.21, *p* = 0.01).

Pooled C data correlated negatively with contrast at 6λ (R^2^ = 0.36, *p* < 0.001) and 2λ (R^2^ = 0.33, *p* < 0.001), indicating less positive Criterion as contrast increases. The ANOVA on CC (change in Criterion) didn’t reveal neither an effect of Stimulation (6λ: F_(1,18)_ = 1.57, *p* = 0.23, η^2^_p_ = 0.08; 2λ: F_(1,18)_ = 0.002, *p* = 0.96, η^2^_p_ < 0.001) nor of the interaction between Stimulation × Contrast Levels: (6λ: F_(6,108)_ = 0.74, *p* = 0.62, η^2^_p_ = 0.039; 2λ: F_(6,108)_ = 2.17, *p* = 0.051, η^2^_p_ = 0.1), indicating that the stimulation did not affect the Criterion.

As Figs. 3 and 5 show, not only tRNS reduced either facilitation or suppression at 6 and 2λ respectively but, for low contrast values, the effect of tRNS resulted into an inversion of SC sign, in both λ sessions. That is, positive SC, at 6λ, turned into negative whereas negative SC, at 2λ, became positive. This was confirmed by one-tail t-tests based on the null hypothesis of 0 SC (Table 2).

**Table 2:** One-tail t-test to compared whether SC in some contrast levels is significantly different from zero value (no modulation effect).

Configuration	Contrast	Modulation	t	*p*
6λ	0.0028	−0.39	−2.50	0.011
	0.0039	−0.26	−1.65	0.059
2λ	0.0030	+0.42	1.76	0.047
	0.0058	+0.44	2.35	0.015

### Control Experiments results

Pooled *d*’s obtained in the control experiments correlated positively with contrast, both at 6λ (R^2^ = 0.46, *p* < 0.001) and 2λ (R^2^ = 0.47, *p* < 0.001), indicating higher sensitivity as contrast increases. The ANOVA didn’t show a significant effect of Stimulation on SC, neither at 6λ (F_(1,14)_ = 0.67, *p* = 0.43, η^2^_p_ = 0.046) nor at 2λ (F_(1,14)_ = 1.83, *p* = 0.20, η^2^_p_ = 0.12). The interaction between Stimulation × Contrast Levels was also not significant, either at 6λ (F_(6,84)_ = 1.21, *p* = 0.69, η^2^_p_ = 0.044) or at 2 λ (F_(6,84)_ = 0.50, *p* = 0.80, η^2^_p_ = 0.035).

Pooled *C* data correlated negatively with contrast at 6λ (R^2^ = 0.25, *p* < 0.001) and 2λ (R^2^ = 0.32, *p* < 0.001), indicating less positive Criterion as contrast increases. The ANOVA on CC revealed neither the effect of Stimulation (6λ: F_(1,14)_ = 0.44, *p* = 0.51, η^2^_p_ = 0.032); 2λ: F_(1,14)_ = 0.1, *p* = 0.92, η^2^_p_ = 0.001) nor of the interaction between Stimulation × Contrast: (6λ: F_(6,84)_ = 0.65, *p* = 0.69, η^2^_p_ = 0.044; 2λ: F_(6,84)_ = 0.5, *p* = 0.8, η^2^_p_ = 0.035), indicating that the stimulation did not affect the Criterion.

## Discussion

In the present study we investigated the tRNS effect on either the target contrast gain or on the relative strength of E or I lateral interactions between the target and collinear flankers in a lateral masking configuration. As expected, the effect of the flankers in the control conditions with sham stimulation, as reflected by SC (*d*’_collinear_ - *d*’_single_) (see Figs. 3 and 5, black broken lines), was either facilitatory in Experiment 1, where flankers were at medium distance from the target (with SC values > 0) or inhibitory in Experiment 2, where target-to-flanker separation was short (with SC values < 0), particularly at medium-high contrast levels. tRNS modulated both these effects in a specific way.

### tRNS reduces facilitation in the 6λ configuration

As Fig. 2 shows, tRNS produced a contrast gain for the single target, as reflected by higher *d*’ obtained in the session with active stimulation than in the session with Sham. Although the difference between the condition with and without electrical stimulation for the collinear stimulus is negligible, it contributes to the global effect represented in Fig. 3. In fact, the SC shows a clear reduction of facilitation by the stimulation: this suggests that the two opposite effects added to produce the SC. SC is therefore more conspicuous than that we would expect on the basis of the effect of tRNS on the collinear configuration, since most of the SC effect results from contrast enhancement by stimulation in the single target configuration. The possibility that the effect of stimulation in the two configurations is opposite may account for the relevant result, discussed later, of a switch of SC from positive to negative at low contrasts.

### tRNS reduces inhibition in the 2λ configuration

The effect of tRNS at 2λ is shown in Fig. 4. The stimulation does not affect *d*’ when the target is presented isolated (single). Since it is well known that the stimulation has little effect with well visible targets tRNS was expected to increase contrast gain at low but not high contrast. In the collinear condition, the tRNS increased sensitivity: the increased *d*’ occurred, in particular at the levels of contrast of 0.0058 and 0.3. This selective effect of tRNS is clearly confirmed by the SC data (Fig. 5), showing that tRNS reduced negativity of SC at these low and high contrast values. Note that, as we will discuss in the next paragraph, at low contrasts SC inverts polarity.

### tRNS inverts the lateral interaction effect

It has been suggested that the facilitation/suppression of the signal by lateral interactions are the result of the balance between E and I lateral interactions^1–8^. Our data seem to indicate that tRNS perturbs this balance. With a target-to-flanker distance of 6λ, not only tRNS reduced flankers facilitation (Fig. 3, see also the figures in the supplementary information) but, at low contrast levels, it changed the sign of SC from positive to negative. Note that this effect of the tRNS on SC is mainly due to an increase of *d*’ by tRNS in the single target configuration and also, to a lesser extent, to a decrease of *d*’ by tRNS in the flanker configuration. With a target-to-flanker distance of 2λ (Fig. 5) the tRNS modulation not only consists in a reduced suppression by the flankers but also, at lower levels of contrast, tRNS turns suppression into facilitation.

These results indicate a dissociation of the tRNS effect within the range of contrasts levels, relatively low, shared by the two target-to-flanker distances. This strongly suggests that the effect consists of a modulation of contextual influences, and not simply of the local detection mechanism.

### The effect of the Criterion

If tRNS effect reflects a genuine modulation of visual sensitivity, no difference in the Criterion obtained in the sham and tRNS sessions should be highlighted, regardless of whether the flankers were present or not. Figure 6 shows the Criterion obtained as a function of contrast in Experiment 1 and 2 where stimulation was delivered to the occipital lobe. Clearly, the Criterion was more conservative (positive) with single target but, as expected, this effect decreased when the target was more detectable at high contrasts. With both flanker separations, at high levels of contrast there is a small change of Criterion polarity, suggesting an increase of false alarms^47^. Importantly, there was no effect of tRNS, regardless of Criterion polarity, confirming the hypothesis of a selective effect of stimulation on visual coding mechanisms and the way they are modulated by contextual influences.

### tRNS administered to a control region has no effect

When tRNS was delivered, as a control, over the forehead, with the other electrode placed over the vertex (Cz) the stimulation had no effect at all on SC and on the CC. This suggests a genuine effect of tRNS on visual coding and contextual influence mechanisms.

### tRNS dependent modulation of E/I balance interpretation

To sum up, behavioral data showed an increase of *d*’ by the flankers at 6λ and the decrease of *d*’ at 2λ, as reflected into a positive and negative SC respectively. These results are consistent with the finding that the flankers facilitate target detection at medium λ and low contrast whereas they inhibit target detection at short λ and relatively high contrast^1–8^.

It has been suggested that the effect of flankers occurs because low contrast targets and large target-to-flankers separations promote activation of E lateral interactions between target and flankers, whereas relatively high contrast targets and short target-to-flankers separation are appropriate for activating lateral interactions or the summation of target and flankers within the target receptive field^1–8,41,48^. It should be noted that the highest facilitation with the large separation is found for low contrasts (ranging from 0.0055 to 0.015 Michelson contrast), whereas for the same contrast values the effect of flankers was negligible with short distance. This suggests that target-to flanker distance plays the most relevant role in determining the polarity of contrast effects due to flankers in human observers.

The neurophysiological mechanism accounting for the dissociation in the contextual influences effects has received great attention. According to it, contrast detection tasks are mediated by the activation of E and I subpopulations of neurons in a cortical column, with the ratio between E and I activation increasing as a consequence of two inputs: stimulus contrast (thalamic input) and the lateral input biased versus excitation^3,4,48–51^.

We suggest that tRNS might perturb E/I balance. The way tRNS produces this effect has been associated to the way stochastic resonance mechanisms operate^32,38,39^. tRNS consists of random frequency stimulation that induces random activity into the system; this activity acts as a pedestal to boost the activation of weakly stimulated neurons. When the input signal is too weak and produces subthreshold neural response, tRNS mediates a cooperation between signal and optimal visual noise, with the result of input enhancement, selectively for subthreshold but not suprathreshold response^39^.

As suggested by the concept of stochastic resonance^38,52^ the input at the threshold level can be better processed within an optimum level of noise compared to without noise. In this framework, the tRNS induced noise, serves as a pedestal to increase the sensitivity of the neurons to a given range of weak inputs, and the final effects are related to the functional activation induced by the state of the system. Importantly this result is corroborated by *in vitro* electrophysiological data^37^ endorsing the hypothesis that electrical RNS of neurons induces facilitation of sodium channels current, at an optimum level of noise for short-term application, via an excitability increase of the stimulated neural system.

We suggest that the modulation by tRNS via stochastic resonance mechanism could account for our three main results: i) tRNS affects sensitivity for the single target only at the low contrasts levels ii) the effect of tRNS on the collinear flankers occurs at both separations and consists in an overall reduction of facilitation with 6λ and a more selective reduction of inhibition with 2λ. iii) At both separations the tRNS inverts the polarity of contextual influences at the lowest levels of contrasts used with the two separations: whereas at 2λ the tRNS turns inhibitory contextual influences into facilitatory, at 6λ tRNS does the opposite.

The neural mechanism accounting for the tRNS-dependent increase of perceived contrast of the single target when it is low (at 6λ) might rely on the evidence that tRNS generally boosts weak neural input^32,36,53,54^. Given that E/I ratio due to thalamic input increases with the contrast of the isolated target, the ratio would be expected to be higher at 2 than 6λ. As a consequence, the facilitation of the isolated target resulting from the increase of neural excitability produced by tRNS, should occur where the E/I ratio is low, i.e., at 6λ. In these conditions, we would expect, as we found, an increase of contrast gain for the low contrast target. With a target of high contrast (Experiment 2) the weight of E and I is strongly biased towards E and tRNS would be ineffective in increasing contrast sensitivity for the isolated target. Our results support the hypothesis of an effect of tRNS based on the modulation E/I ratio.

Moreover, to fit the action of tRNS with that of a stochastic resonance mechanism we have to accept that both the response to the target (by thalamic input) and to the flankers are modulated by a low/appropriate level of noise. That is, whenever the neural response is weak, it is boosted by the tRNS.

This hypothesis is compatible with the weak tRNS effect obtained when the flankers are 6λ apart from the target. At these target-to-flanker separations, as our (Fig. 2) and previous data show, lateral input is biased towards excitation^1,2,7^. Given an excess of lateral excitation the inhibition would be comparatively weak and therefore, we would expect tRNS to boost inhibitory connection. Indeed, we found that tRNS slightly reduced facilitation by the flankers.

At 2λ, the expected effect of the tRNS on lateral input is opposite. The stronger activation of inhibitory lateral input when close flankers are present would reduce the E/I ratio in the cortical column activated by the target. Based on this assumption, by boosting the weaker, excitatory lateral input, the tRNS would produce a reduction of inhibition.

In favor of the modulation of lateral E/I input by tRNS is our third result: the E/I balance perturbation result discussed in paragraph “*tRNS inverts the lateral interaction effect***”**. It showed that even when the target contrast matches in the two λ distances and therefore there is no change in thalamic input, there is still a boosting effect of lateral interactions by tRNS, but in opposite direction at the two separations.

It should be remarked that the effects of tRNS, at large separations, may be compatible with the way the polarity of lateral interactions depends on the target contrast in normal vision. It has been shown in previous studies that for target contrast ranging from low to very low with respect to that of the flankers, the lateral input switches from facilitation to inhibition^41,55^. Such a contextual modulation contrast-dependent could explain both the reduction of facilitation by tRNS and the switch from facilitation to inhibition at very low contrast (Fig. 3). This model however does not explain the tRNS-dependent reduction of inhibition by the flankers at 2λ for two reasons: first this mechanism only works for a low contrast range (the contrast range used in this study at 2λ is higher than 6λ) and second tRNS should have had increased the perceived contrast of the isolated target not only at 6λ but also at 2λ, at corresponding contrast levels, but it did not. Therefore, at both separations lateral interaction modulation by tRNS should be called into cause since the two separations produce opposite modulatory contextual effect by tRNS at corresponding contrast level. At 6λ only, the modulation of lateral interactions may also depend on perceived contrast for the isolated target.

A final comment should be made on the evidence (Fig. 6) that in Experiment 2 but not 1 tRNS sets observers’ Criterion to a more conservative value (not significantly). However, it is unlikely that this affect the way tRNS affects lateral interactions since sensitivity does not change^47^.

To sum up, we have shown a dissociated of tRNS effect: tRNS can either reduce or increase the modulation that collinear flankers exert on contrast sensitivity of a low contrast target. Overall, tRNS increased the efficiency of whatever lateral interactions are weak: excitatory at short target-flanker separations, inhibitory at medium separations. The dissociation results from a partially complementary effect of tRNS. At large and facilitatory target-to-flanker separations tRNS increases contrast sensitivity for the low contrast target leading, as it occurs in normal vision and produces a modulation of lateral input towards reduced facilitation or to a switch from facilitation to inhibition. When the target-to-flanker separation is short and inhibitory and a target contrast is high, tRNS affects directly the inhibitory lateral interactions reducing its strength.

In conclusion, the evidence that tRNS modulates intracortical lateral interactions at low level of central visual processing in the human brain can have relevant clinical consequences. tRNS might be used to boost the effect of visual training in restoring lateral intracortical connections in V1 when these are made inefficient by visual disorders such as amblyopia and macular degeneration^17–24^.

## Supplementary information

Supplementary figures
