# Supplementary material for: Excitatory and inhibitory lateral interactions effects on contrast detection are modulated by tRNS
Source: Sci Rep. 2019 Dec 17;9:19274. doi: 10.1038/s41598-019-55602-z (PMC6917720; doi:10.1038/s41598-019-55602-z)
Supplement: Supplementary file 1 — Supplementary figures [file 41598_2019_55602_MOESM1_ESM.docx]

Title: Excitatory and inhibitory lateral interactions effects on contrast detection are modulated by tRNS

**Battaglini L. ^1,2*^, Contemori G.^1,2,3^, Fertonani A.^4^, Miniussi C.^4,5^, Coccaro A.^1,2^, Casco C. ^1,2^**

^1^Department of General Psychology, University of Padova, Padova, Italy

^2^Neuro.Vis.U.S. Laboratory, University of Padova, Padova, Italy

^3^Université de Toulouse-UPS, Centre de Recherche Cerveau et Cognition, Toulouse, France

^4^Cognitive Neuroscience Section, IRCCS Istituto Centro San Giovanni di Dio Fatebenefratelli, Brescia, Italy

^5^Center for Mind/Brain Sciences - CIMeC, University of Trento, Rovereto, Italy

***Corresponding authors:**

*Contact address:*

Luca Battaglini

Department of General Psychology

University of Padova

Via Venezia, 8

35131 Padova (Italy)

E-mail: [luca.battaglini@unipd.it](mailto:)

Phone: +39 8276149

This supplementary material contains Figure 7 and 8 that show CSs (*d*’_collinear_ - *d*’_single_) obtained in Experiment 1 (**6λ)** and 2 (**2λ)**. In these Figures are represented differences in pairs of conditions that are not shown in Figure 3 and 5.


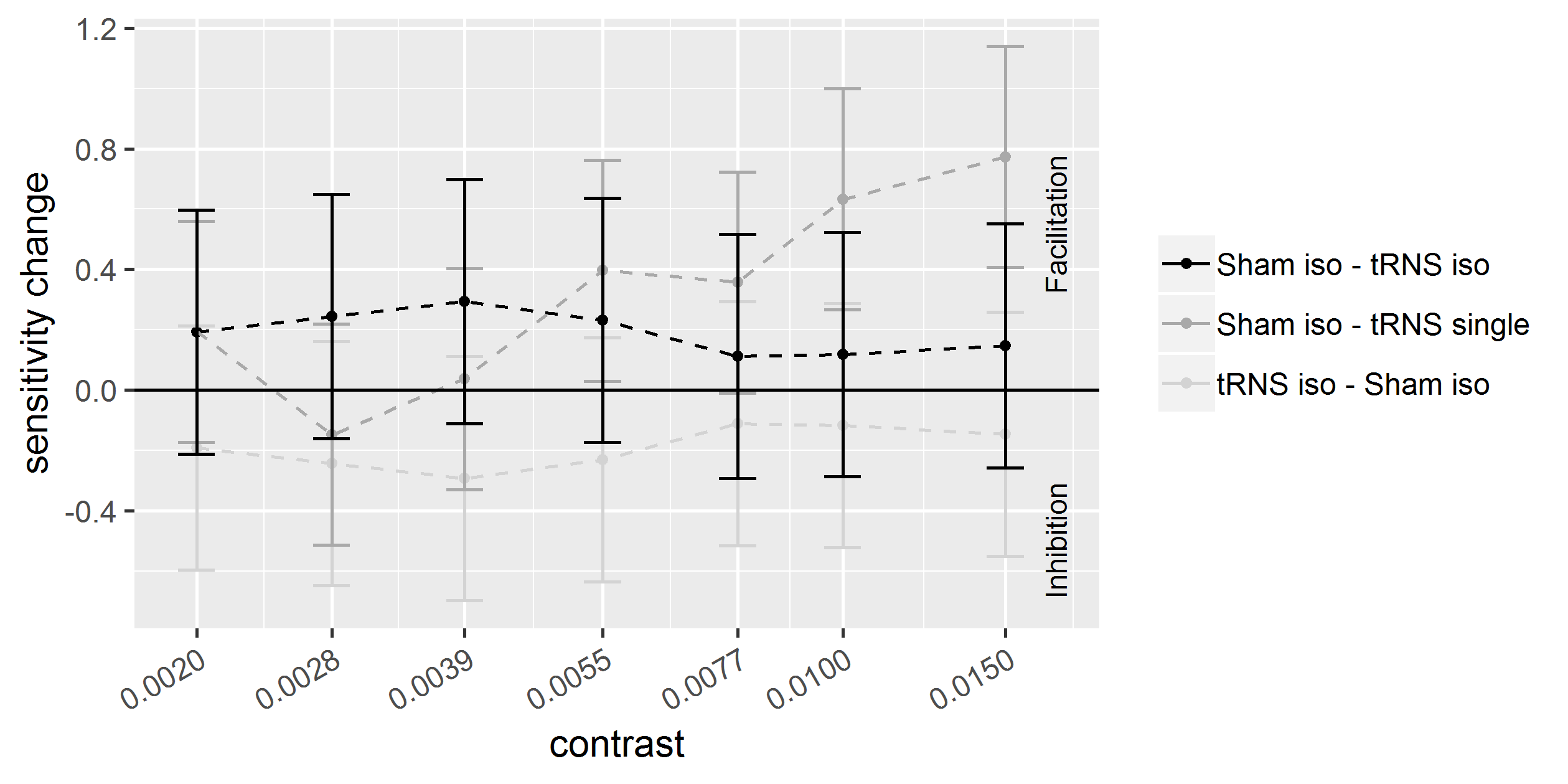


Figure 7. Sensitivity changes (SC), referring to the difference between *d*’ obtained in the collinear and single target (*d*’_collinear_ - *d*’_single_), are plotted as a function of target contrast with flankers at a distance of **6λ.** Positive values represent facilitation by collinear flankers whereas negative values represent inhibition. Solid bars indicate confidence interval (95%).


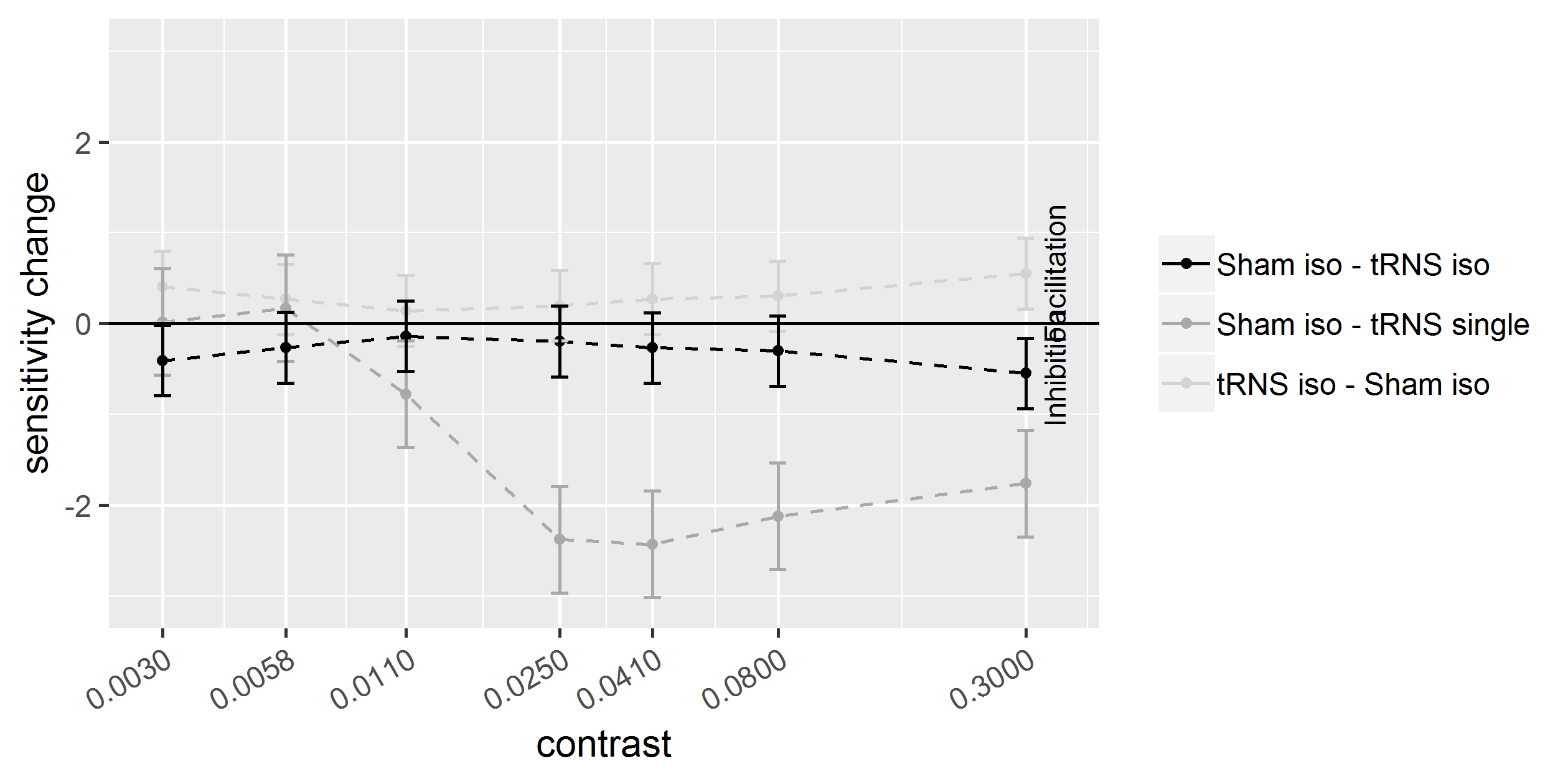


Figure 8. Sensitivity changes (SC), referring to the difference between *d*’ obtained in the collinear and single target (*d*’_collinear_ - *d*’_single_), are plotted as a function of target contrast with flankers at a distance of **2λ.** Positive values represent facilitation by collinear flankers whereas negative values represent inhibition. Solid bars indicate confidence interval (95%).
